# Downregulated METTL14 Expression Correlates with Breast Cancer Tumor Grade and Molecular Classification

**DOI:** 10.1155/2020/8823270

**Published:** 2020-10-20

**Authors:** Xiao-Fang Dong, Yan Wang, Bi-Fei Huang, Gui-Nv Hu, Jun-Kang Shao, Qian Wang, Chih-Hsin Tang, Chao-Qun Wang

**Affiliations:** ^1^Department of Medical Oncology, Affiliated Dongyang Hospital of Wenzhou Medical University, Dongyang, Zhejiang, China; ^2^Department of Pathology, Affiliated Dongyang Hospital of Wenzhou Medical University, Dongyang, Zhejiang, China; ^3^Department of Surgical Oncology, Affiliated Dongyang Hospital of Wenzhou Medical University, Dongyang, Zhejiang, China; ^4^Graduate Institute of Basic Medical Science, China Medical University, Taichung, Taiwan; ^5^Department of Pharmacology, School of Medicine, China Medical University, Taichung, Taiwan; ^6^Department of Biotechnology, College of Health Science, Asia University, Taichung, Taiwan

## Abstract

It is unclear whether the methyltransferase-like 14 (METTL14) protein promotes or suppresses cancer growth. We examined the association between METTL14 expression, cancer progression, and patient prognosis in a total of 398 breast cancer tissue specimens. Significantly fewer cancer tissue specimens compared with normal breast tissue expressed high levels of METTL14 (52.8% vs. 75.0%). METTL14 expression was negatively associated with tumor grade and positively associated with patient age, estrogen, and progesterone receptor status. High METTL14 expression was more common in luminal A and luminal B tissue (75.9% and 60.8%, respectively), compared with human epidermal growth factor receptor 2- (HER2-) enriched and triple-negative breast cancer (TNBC) samples (38.2% and 18.6%, respectively). In multiple logistic regression analysis, independent predictors of METTL14 expression in breast cancer included higher tumor grade (odds ratio (OR) = 0.494, 95% confidence interval (CI): 0.289–0.844; *P* = 0.010), TNBC subtype (OR = 0.109, 95% CI: 0.054–0.222; *P* < 0.001), and HER2-enriched subtype (OR = 0.298, 95% CI: 0.156–0.567; *P* < 0.001). No clear relationship was observed between patient prognosis and METTL14 expression. It appears that downregulated METTL14 expression in breast cancer is associated with tumor grade and molecular classification.

## 1. Introduction

Breast cancer is the leading cause of cancer-related death among women [1]. Breast cancer is a biologically diverse disease, reflected by different molecular subtypes that have different biological behaviors and clinical treatment options [2–4]. The different subtypes include luminal A and luminal B, human epidermal growth factor receptor 2- (HER2-) enriched, and triple-negative or basal-like; immunohistochemistry (IHC) can assess standard biomarkers (estrogen receptor (ER), progesterone receptor (PR), and HER2) as well as Ki-67 status (a marker of cell proliferation) [2, 5]. Triple-negative breast cancer (TNBC) has a more aggressive clinical course and poorer prognosis than other types of breast cancer, and currently there is no effective treatment other than chemotherapy [6–9]. Thus, studying the molecules related to the occurrence and progression of breast cancer and their clinical significance is of great significance for guiding the treatment of breast cancer, especially TNBC.

The catalytic protein methyltransferase-like 14 (METTL14) participates in mediate cellular *N*^6^-methyladenosine (m^6^A) deposition [10] and is closely related to the occurrence and development of malignant tumors [11–23]. METTL14 plays a complex role in malignant tumors: low levels of METTL14 expression are found in hepatocellular carcinoma [13], colorectal cancer [14, 16, 17], bladder cancer [15], gastric cancer [18], childhood ETV6/RUNX1-positive acute lymphoblastic leukemia [19], and papillary thyroid carcinoma [20], where METTL14 functions as a tumor suppressor [13–18], whereas levels of METTL14 expression are high in pancreatic cancer [21, 22] and acute myeloid leukemia [23], where this protein functions as a tumor promoter. In breast cancer, contrasting findings have been reported from analyses of the expression and function of METTL14. In one study, significantly decreased METTL14 expression was observed in patients with breast cancer compared with healthy controls, and overexpression of METTL14 inhibited breast cancer cell viability, colony formation, and migration [24]. Conversely, in another study, significantly upregulated METTL14 expression was observed in breast cancer tissue compared with normal tissue, and METTL14 overexpression enhanced the migration and invasion capacities of breast cancer cells [25], while other researchers have reported significantly upregulated levels of METTL14 and m^6^A in breast cancer cells and tissues, and the modulation of METTL14 by long noncoding RNA LINC00942 (LNC942) promoted breast cancer initiation and progression [26]. Thus, further research is needed to better understand the expression and function of METTL14 in breast cancer. This study performed an IHC analysis of METTL14 expression in breast cancer tissue samples obtained from 398 Chinese Han women, to clarify the expression of METTL14 in breast cancer and its clinicopathological and prognostic significance.

## 2. Materials and Methods

### 2.1. Patients and Tissue Samples

Breast cancer tissue samples were obtained from 398 untreated Chinese Han women aged 24–90 years (median 50 years) who underwent surgery in the Affiliated Dongyang Hospital of Wenzhou Medical University (Dongyang, Zhejiang, China) between 2007 and 2019. Twenty-four samples of adjacent normal breast tissue were also obtained following surgical resection. A pathohistological diagnosis was made according to the breast tumor classification criteria of the World Health Organization [27]. Histological grading was based on the Scarff-Bloom-Richardson system [28]. According to ER, PR, HER2, and Ki-67 status, the samples were classified into 4 subtypes [2, 5]: luminal A (ER^+^/PR^+^ [≥20%]/HER2^–^, Ki67 < 14%); luminal B, containing hormone receptor-positive cases that did not meet the conditions of luminal A; HER2-enriched (ER^–^, PR^–^, and HER2^+^); or TNBC (ER^–^, PR^–^, and HER2^–^). Follow-up information was available for 228 breast cancer patients with a median follow-up time of 60 months (range, 6–70 months). The Medical Ethics Committee of the Affiliated Dongyang Hospital of Wenzhou Medical University approved this study. All study methods satisfied the relevant guidelines and regulations issued by the Affiliated Dongyang Hospital of Wenzhou Medical University.

### 2.2. Tissue Array Preparation

We followed the methods described by Wang et al. [29]. In brief, the Quick-Ray^®^ UT-06 (Unitma Co., Ltd., Seoul, Korea) tissue microarray system and the Quick-Ray premade recipient block (UB-06) wax model were used to prepare tissue specimens (1 mm in diameter). Two representative sites from each breast cancer tissue sample were selected for sampling.

### 2.3. IHC Analysis

IHC staining of paraffin-embedded tissue sections used the Envision System (Dako, Glostrup, Denmark), as described previously [5, 30]. Primary antibodies consisted of anti-METTL14 mouse monoclonal antibody (clone CL4252, diluted at 1 : 1000; Abcam, Cambridge, England), ready-to-use anti-ER rabbit monoclonal antibody (clone SP1, Dako), ready-to-use anti-PR mouse monoclonal antibody (clone PgR636, Dako), HercepTest (Dako), and ready-to-use anti-Ki-67 mouse monoclonal antibody (clone MIB-1, Dako).

### 2.4. Assessment of Staining

Each entire section was scanned and scored independently by 2 pathologists. The intensity of nuclear staining for METTL14 was assessed in breast tissue. Staining intensity was scored on a 4-point scale from 0 (negative) to 1 (weak), 2 (moderate), or 3 (strong). High METTL14 expression was defined as a staining intensity of positive invasive cancer cells of 2 or 3 [31]. A case was considered to be ER- or PR-positive if the percentage of positive invasive cancer cells (nuclear staining) was ≥1% [32]. HER2 status was determined by the 2018 American Society of Clinical Oncology/College of American Pathologists guidelines for HER2 testing in breast cancer [33].

### 2.5. Patient Follow-Up

We followed the methods of Wang et al. [29]. In brief, each patient was followed up postoperatively by telephone call and thereafter at 6 monthly hospital appointments; follow-up was discontinued in the event of the patient's death. A diagnosis of local breast cancer recurrence was made by clinical or histology results. Relapse-free survival (RFS) was defined as the time from surgery to relapse/metastasis; overall survival (OS) was the time from surgery to death (excluding non-tumor-related deaths).

### 2.6. Flow Diagram

**Figure 1:**
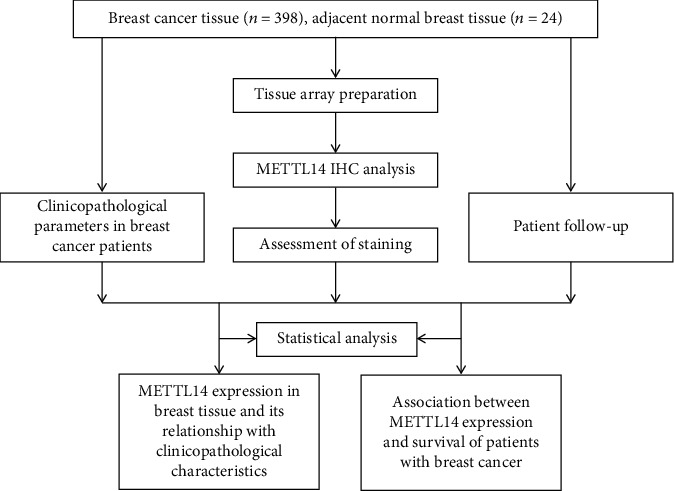
Flow diagram of the study methodology.

A flowchart of the study methodology is shown in Figure 1.

### 2.7. Statistical Analysis

Statistical analyses were conducted using SPSS software version 19.0 (SPSS Inc., Chicago, IL, USA). Between-group differences were compared using Pearson's chi-square test for qualitative variables. The relative risk was expressed by odds ratios (ORs) and their 95% confidence intervals (CIs). The independent correlation factor of METTL14 expression in breast cancer was assessed by multiple logistic regression analysis. RFS and OS rates were estimated by the Kaplan-Meier method and compared using log-rank testing. *P* < 0.05 was considered to be statistically significant.

## 3. Results

### 3.1. METTL14 Expression in Breast Tissue and Its Relationship with Clinicopathological Characteristics

**Figure 2:**
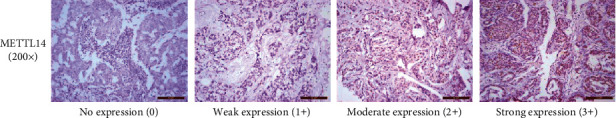
METTL14 expression levels in breast cancer patients. Breast cancer tissue specimens were immune-stained with anti-METTL14 antibody, photographed using an optical microscope, and scored from 0 (negative) to 3 (strong) for nuclear staining intensity of METTL14 expression.

Around half of the breast cancer tissue specimens (52.8%, 210/398) expressed high METTL14 expression, compared with 75.0% (18/24) of normal breast tissue specimens (Figure 2); the between-group difference was significant (*P* = 0.034) (Table 1).

As shown in Table 2, high METTL14 expression was identified in 64.2% (174/271) of grade I–II tumor tissue and in 28.3% (36/127) of grade III tumor tissue; the between-group difference was highly significant (*P* < 0.001). In younger-aged patients (≤35 years) and in those whose tumors were ER- or PR-negative, rates of high METTL14 expression were 31.8% (7/22), 28.6% (48/168), and 39.2% (82/209), respectively, which were significantly lower than the corresponding rates among older-aged patients (>35 years; 54.0% [203/376]), ER-positive tissue (70.4%, 162/230), and PR-positive tissue (67.7%, 128/189) (*P* = 0.043, *P* < 0.001, and *P* < 0.001, respectively). Among the 4 molecular subtypes, TNBC was associated with the lowest rate of high METTL14 expression (18.6%, 18/97), compared with rates of 43.3% (29/67) for HER2-enriched tumors, 60.8% (59/97) for luminal B, and 75.9% (104/137) for luminal A tumors (*P* < 0.001). In multiple logistic regression analysis, independent predictors of METTL14 expression in breast cancer included higher tumor grade (odds ratio (OR) = 0.494, 95%confidence interval (CI): 0.289–0.844; *P* = 0.010), the presence of the TNBC subtype (OR = 0.109, 95% CI: 0.054–0.222; *P* < 0.001), and the presence of the HER2-enriched subtype (OR = 0.298, 95% CI: 0.156–0.567; *P* < 0.001).

### 3.2. No Association between METTL14 Expression and Survival of Patients with Breast Cancer

**Figure 3:**
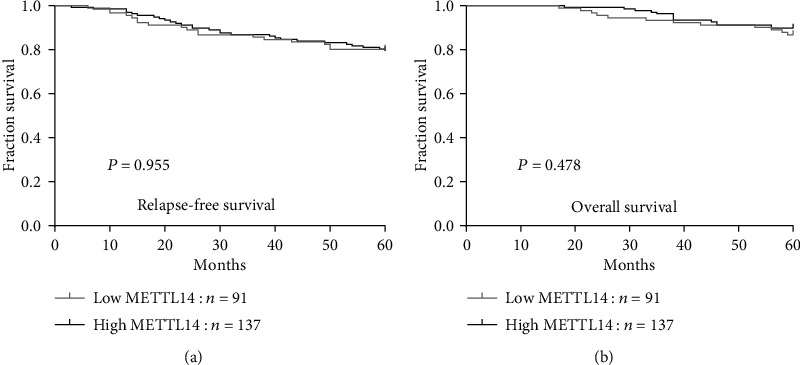
METTL14 expression is not associated with the survival of patients with breast cancer. The associations of METTL14 expression with relapse-free survival (a) and overall survival (b).

When we examined the data for potential associations between levels of METTL14 expression and survival rates in a cohort of 228 breast cancer patients, 5-year RFS and OS rates were 79.8% and 88.6%, respectively. As shown in Figure 3, no clear associations were observed between METTL14 expression and survival. The 137 patients with high levels of METTL14 expression had a mean RFS of 54.0 months and an estimated 5-year RFS rate of 79.6%; corresponding values in the 91 patients whose tumors expressed low levels of METTL14 were 53.2 months and 80.2%, respectively (*P* = 0.955; Figure 3(a)). Mean OS was 57.9 months (with an estimated 5-year OS rate of 89.8%) in the patients with high levels of METTL14 expression and 57.0 months (with an estimated 5-year OS rate of 86.8%) in those with low levels of METTL14 (*P* = 0.478; Figure 3(b)).

## 4. Discussion

Several studies have reported that METTL14 plays a complex role in the occurrence and development of malignant tumors [11–23]. For example, in hepatocellular carcinoma, RFS is adversely affected by downregulated levels of METTL14 expression [13], while a reduction in m^6^A mRNA methylation caused by the *METTL14* loss-of-function R298P mutation increases the proliferation and tumorigenicity of endometrial cancer cells [34]. In contrast, METTL14 is highly expressed in acute myeloid leukemia cells and promotes leukemogenesis via m^6^A modification [23]. Findings from three investigations into METTL14 expression and function in breast cancer are controversial, with one study showing that METTL14 acts as a tumor suppressor [24], while the other two show that METTL14 promotes cancer growth [25, 26].

Our findings revealed that METTL14 expression was significantly downregulated in women with higher-grade tumors. Since such tumors are known to be more aggressive and to have a poor prognosis [28], this finding indicates that the downregulation of METTL14 expression may be closely related to highly invasive breast cancer and a poor prognosis. Our results also revealed significantly decreased levels of METTL14 expression in ER- and PR-negative disease, which was closely and negatively correlated with hormone receptor expression. Furthermore, evidence has shown that overexpression of METTL14 in the ER- and PR-negative breast cancer cell line MDA-MB-231 inhibits its proliferation and migration ability [24]. Since hormone receptor-negative breast cancer patients are unsuitable candidates for endocrine therapy, further research should examine whether these patients may benefit from the exogenous overexpression of METTL14. We also found in this study that METTL14 expression was significantly decreased in younger-aged women (≤35 years), who have been shown to have more aggressive breast cancer and a worse prognosis [35, 36]. Our analysis showed that having higher-grade disease, the TNBC subtype or the HER2-enriched subtype is independently correlated with low METTL14 expression in breast cancer. These results suggest that METTL14 expression in breast cancer is correlated with tumor grade and molecular classification, although our survival analysis failed to reveal any association between METTL14 expression and survival. More research is needed to determine how METTL14 expression affects the prognosis of breast cancer patients.

TNBC disease is hormone receptor-negative and HER2-negative, is associated with a younger age at diagnosis, a higher tumor grade, a more aggressive clinical course, and poorer prognosis than other types of breast cancer, and is not amenable to endocrine therapy or HER2-targeted agents, such as trastuzumab [6–9]. All of these factors highlight the need for new treatment options for TNBC. Our findings showing that METTL14 expression was the lowest in TNBC compared with other subtypes of breast cancer suggests that it may be worth targeting this protein in the treatment of patients with TNBC.

## Figures and Tables

**Table 1:** METTL14 expression in breast tissue specimens.

Group	No.	METTL14 expression	
		Low expression, *n* (%)	High expression^†^, *n* (%)
Normal tissue	24	6 (25.0%)	18 (75.0%)
Cancerous tissue	398	188 (47.2%)	210 (52.8%)^∗^

^†^High METTL14 expression was defined as a nuclear staining intensity of positive invasive cancer cells of 2 or 3 [17]. ^∗^*P* < 0.05 vs. normal breast tissue.

**Table 2:** Association of METTL14 expression with clinicopathological parameters in breast cancer patients.

Parameters	No. of patients	High METTL14 expression, *n* (%)	*p* value
Age (years)			
≤35	22	7 (31.8%)	0.043
>35	376	203 (54.0%)	
Tumor size (cm)			
≤2	176	99 (56.3%)	0.365
2–5	201	102 (50.7%)	
>5	21	9 (42.9%)	
Lymph node metastases			
No	203	99 (48.8%)	0.103
Yes	195	111 (56.9%)	
Tumor grade			
I–II	271	174 (64.2%)	<0.001
III	127	36 (28.3%)	
Tumor stage			
I	103	59 (57.3%)	0.552
II	201	102 (50.7%)	
III	94	49 (52.1%)	
IV	0	0 (0.00)	
Estrogen receptor			
Negative	168	48 (28.6%)	<0.001
Positive	230	162 (70.4%)	
Progesterone receptor			
Negative	209	82 (39.2%)	<0.001
Positive	189	128 (67.7%)	
HER2 expression			
Negative (0–1^+^)	191	93 (48.7%)	0.095
Equivocal (2^+^)	109	67 (61.5%)	
Positive (3^+^)	98	50 (51.0%)	
Molecular classification			
Luminal A	137	104 (75.9%)	<0.001
Luminal B	97	59 (60.8%)	
HER2-enriched	67	29 (43.3%)	
TNBC	97	18 (18.6%)	

^∗^High METTL14 expression was defined as a nuclear staining intensity of positive invasive cancer cells of 2 or 3 [17]. Abbreviations: HER2: human epidermal growth factor receptor 2; TNBC: triple-negative breast cancer.

## Data Availability

All data generated or analyzed during this study are included in this published article.

## Abbreviations

METTL14: Methyltransferase-like 14
ER: Estrogen receptor
PR: Progesterone receptor
HER2: Human epidermal growth factor receptor 2
RFS: Relapse-free survival
OS: Overall survival
IHC: Immunohistochemistry
TNBC: Triple-negative breast cancer.
